# INTEREST GROUP SESSION—FAMILY CAREGIVING: SUPPORTING FAMILY CAREGIVERS WITHIN SYSTEMS OF CARE DELIVERY

**DOI:** 10.1093/geroni/igz038.746

**Published:** 2019-11-08

**Authors:** Jennifer L Wolff, Lynn F Feinberg

**Affiliations:** 1 Johns Hopkins University, Maryland, United States; 2 AARP Public Policy Institute, Washington, District of Columbia, United States

## Abstract

Family and other unpaid caregivers have a foundational role in supporting the health and well-being of older adults with complex health needs and disabilities and the demands imposed on them can be significant. The availability and adequacy of support provided by family and other unpaid caregivers has profound consequences for quality and outcomes of care delivery, but they are not well-supported in treatment decisions and care planning. Given population aging, the shift of long-term services and supports from nursing homes toward community settings, and technological advances that allow patients to be served in the community with higher acuity of care, there is a pressing need to develop systems-level processes to identify, engage, and support family caregivers in systems of care. This symposium will feature 5 presentations that provide novel insight regarding family caregivers’ experience within systems of care. We focus on family caregivers to older adults living in the community and receiving home and community-based services, primary care, or Medicare skilled home health services. Individual presentations will describe 1) differences in access to services and experiences of family caregivers by under-represented minority status; 2) a framework for health systems to include family caregivers as part of health care teams; 3) family caregivers’ capacity to help during the course of Medicare-funded skilled home health care; 4) perceived communication with health professionals, using a validated measure of family caregiver capacity; and 5) the feasibility of implementing a family caregiver screening instrument in primary care.

